# PACAP-38 and PAC1 Receptor Alterations in Plasma and Cardiac Tissue Samples of Heart Failure Patients

**DOI:** 10.3390/ijms23073715

**Published:** 2022-03-28

**Authors:** Dóra Szabó, Zsolt Sárszegi, Beáta Polgár, Éva Sághy, Dóra Reglődi, Tünde Tóth, Zsófia Onódi, Przemyslaw Leszek, Zoltán V. Varga, Zsuzsanna Helyes, Ágnes Kemény, Péter Ferdinandy, Andrea Tamás

**Affiliations:** 1 Heart Institute, Clinical Centre, Medical School, University of Pecs, 7624 Pecs, Hungary; dora0szabo@gmail.com (D.S.); sarszegizsolt@gmail.com (Z.S.); 2 Department of Anatomy, MTA-PTE PACAP Research Team, Centre for Neuroscience, Medical School, University of Pecs, 7624 Pecs, Hungary; dora.reglodi@aok.pte.hu (D.R.); toth.tundi206@gmail.com (T.T.); 3 Szentagothai Research Centre, University of Pecs, 7624 Pecs, Hungary; zsuzsanna.helyes@aok.pte.hu (Z.H.); kemeny.agnes@pte.hu (Á.K.); 4 Department of Medical Microbiology and Immunology, Clinical Centre, Medical School, University of Pecs, 7624 Pecs, Hungary; beata.polgar@aok.pte.hu; 5 Cardiometabolic Research Group, MTA-SE System Pharmacology Research Group, Department of Pharmacology and Pharmacotherapy, Semmelweis University, 1089 Budapest, Hungary; saghy.eva@med.semmelweis-univ.hu (É.S.); onodi.zsofia@med.semmelweis-univ.hu (Z.O.); varga.zoltan@med.semmelweis-univ.hu (Z.V.V.); peter.ferdinandy@pharmahungary.com (P.F.); 6 HCEMM-SU Cardiometabolic Immunology Research Group, Semmelweis University, 1089 Budapest, Hungary; 7 Department of Heart Failure and Transplantology, Cardinal Stefan Wyszyński National Institute of Cardiology, 04-628 Warszawa, Poland; p.leszek@ikard.pl; 8 Department of Pharmacology and Pharmacotherapy, Medical School, University of Pecs, 7624 Pecs, Hungary; 9 Department of Medical Biology, Medical School, University of Pecs, 7624 Pecs, Hungary; 10 Pharmahungary Group, 6720 Szeged, Hungary

**Keywords:** pituitary adenylate cyclase activating polypeptide, heart failure, ischemic/non-ischemic cardiomyopathy, NT-proBNP, cytokines

## Abstract

Pituitary adenylate cyclase activating polypeptide-38 (PACAP-38) is a multifunctional neuropeptide, which may play a role in cardioprotection. However, little is known about the presence of PACAP-38 in heart failure (HF) patients. The aim of our study was to measure the alterations of PACAP-38 like immunoreactivity (LI) in acute (*n* = 13) and chronic HF (*n* = 33) and to examine potential correlations between PACAP-38 and HF predictors (cytokines, NT-proBNP). Tissue PACAP-38 LI and PAC1 receptor levels were also investigated in heart tissue samples of patients with HF. Significantly higher plasma PACAP-38 LI was detected in patients with acute HF, while in chronic HF patients, a lower level of immunoreactivity was observed compared to healthy controls (*n* = 13). Strong negative correlation was identified between plasma PACAP-38 and NT-proBNP levels in chronic HF, as opposed to the positive connection seen in the acute HF group. Plasma IL-1 β, IL-2 and IL-4 levels were significantly lower in chronic HF, and IL-10 was significantly higher in patients with acute HF. PACAP-38 levels of myocardial tissues were lower in all end-stage HF patients and lower PAC1 receptor levels were detected in the primary dilated cardiomyopathy group compared to the controls. We conclude that PACAP-38 and PAC1 expression correlates with some biomarkers of acute and chronic HF; therefore, further studies are necessary to explore whether PACAP could be a suitable prognostic biomarker in HF patients.

## 1. Introduction

Heart failure (HF) is the most common cause of cardiac death, despite several novel diagnostic and therapeutic opportunities. The most common clinically relevant parameters for the evaluation of the severity of HF with reduced ejection fraction (HFrEF) are the (i) left ventricular ejection fraction (EF), (ii) plasma N-terminal pro-brain natriuretic peptide (NT-proBNP) level and (iii) functionally, the New York Heart Association (NYHA) classification [1,2]. However, EF or NT-proBNP may change during treatment and do not always predict prognosis correctly. Therefore, new factors are being investigated in order to serve as predictive biomarkers for HF prognosis [3].

Several factors, such as oxidative stress, cardiomyocyte necrosis, apoptosis and a range of adaptive mechanisms, including neurohumoral imbalance, increased sympathetic activation, increased cytokine release and different pro- and anti-inflammatory factors, play important roles in the pathophysiology and progression of the disease. Earlier studies showed that levels of various pro-inflammatory cytokines (e.g., IL-1 β, IL-2, IL-6, TNFα), chemokines (e.g., monocyte chemotactic protein—MCP-1) and neutrophil-specific chemokines (different CXC chemokines) are altered, which is often strongly correlated with the severity of HFrEF. These factors promote the development of myocardial remodelling with cardiomyocyte apoptosis and enhanced interstitial fibrosis, eventually exacerbating the impairment of left ventricular systolic function [4,5].

Among several cardioprotective factors, pituitary adenylate cyclase activating polypeptide (PACAP) and its specific PAC1 receptor are widely investigated in different models of HF. There are two biologically active forms of PACAP containing 27 (PACAP-27) or 38 amino acids (PACAP-38). More than 90% of the endogenous PACAP exists as PACAP-38 [6]. Anti-apoptotic, anti-ischemic and anti-inflammatory effects of PACAP-38 are well known [7,8,9,10,11,12]. Sano and co-workers were the first to demonstrate the cardioprotective effect of PACAP in 2002 revealing the ability of PACAP to diminish myocardial fibrosis [13]. Based on earlier results, PACAP seems to ameliorate the prognosis primarily in cardiovascular diseases (such as HF and ischemic heart disease), which are propelled by oxidative stress and/or apoptosis [8,9,11,14]. PACAP effectively promotes defense mechanisms in cardiomyocytes against oxidative stress-induced or ischemia/reperfusion-induced apoptosis in cell culture [9,11,14].

Besides cardiac remodeling, neurohormonal activation and necrosis, apoptosis is also involved in the pathomechanism of HF [15,16,17]. The cardioprotective effect of PACAP was proved in several in vitro and animal cardiotoxicity models, which is carried out by different antiapoptotic mechanisms [18,19,20]. Despite these promising experimental data on the protective effects of PACAP in HF, little is known about its alterations and potential relevance in humans. In one of our previous studies conducted on patients with acute ST segment elevation myocardial infarction, we detected significantly higher plasma PACAP-38 levels before the revascularisation compared to the plasma samples collected from patients 4, 24 and 48 h after coronary intervention, as well as samples from healthy controls [21]. Based on these findings, the question arises whether plasma PACAP-38 levels correlate with other well-known HF predictors, such as cytokines or NT-proBNP, which may indicate the severity of HF or the efficacy of therapeutic interventions adequately.

Therefore, the aim of our study was to measure the alterations of plasma PACAP-38 like immunoreactivity (PACAP-38 LI) in acute and chronic HF caused by ischemic or non-ischemic cardiomyopathy compared to age-matched healthy controls. We also examined the potential correlation between PACAP-38 and other HF predictors, such as NT-proBNP, routine laboratory parameters and different cytokines (IL-1 β, IL-2, IL-4, IL-6. IL-10, IFN-γ, TNF-α) measured with Luminex array. Moreover, tissue PACAP-38 LI was also investigated with PACAP-38 ELISA, and PAC1 receptor levels were examined with Western blot techniques in heart tissue samples of patients with end-stage cardiomyopathy compared to healthy controls.

## 2. Results

### 2.1. Comparison of Plasma PACAP-38 Levels in HF Patients and Healthy Control Individuals

The main demographic and clinical parameters of the examined patients and controls are presented in Table 1.

**Figure 1:**
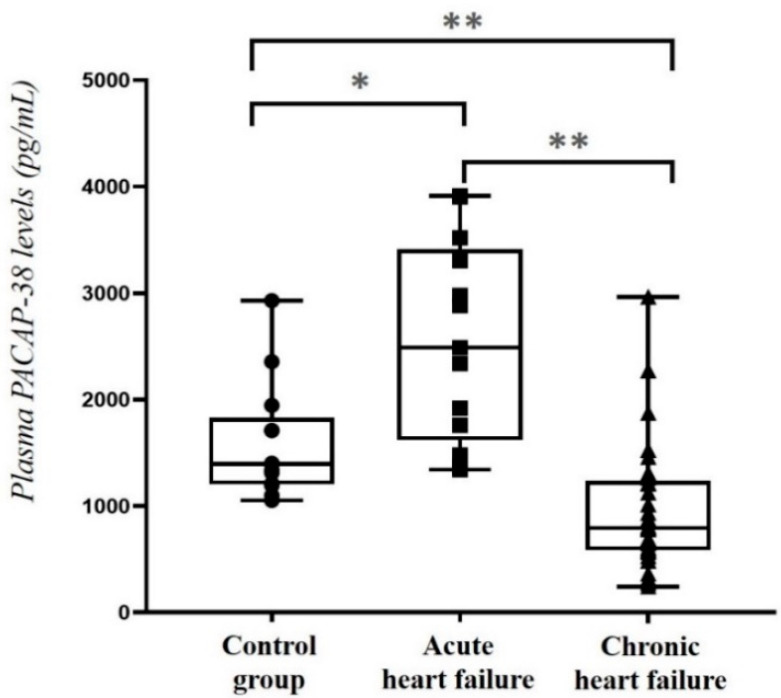
Plasma PACAP-38 levels in acute (decompensated) (*n* = 13) and chronic (compensated) (*n* = 33) heart failure patients compared to the control group (*n* = 13). The box plot diagram represents the interquartile range and median values. The individual values are presented with black dots (control group), squares (acute HF) or triangles (chronic HF). Statistical analysis was performed with one-way ANOVA with Tukey post-hoc test. * *p* < 0.05, ** *p* < 0.001.

First, we examined the plasma PACAP-38 levels of the different groups. Significantly higher plasma PACAP-38 levels were detected in acute HF patients compared to the chronic HF patients (*p* < 0.001) and also to the control group (*p* < 0.05). Furthermore, we detected significantly lower plasma PACAP-38 levels in the chronic HF patients compared to both acute HF group (*p* < 0.001) and the control group (*p* = 0.001) (Figure 1).

### 2.2. Correlation of Plasma PACAP-38 Levels with NT-proBNP Levels in HF Patients

**Figure 2:**
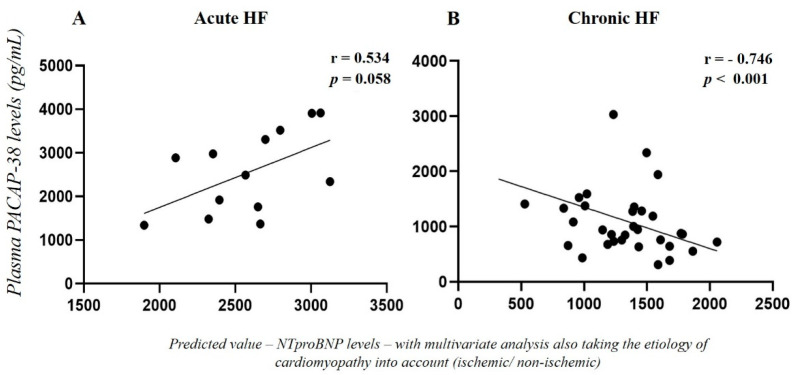
Correlation between the predicted value of NT-proBNP and plasma PACAP-38 levels (pg/mL) in acute (**A**) (*n* = 13) and chronic (**B**) (*n* = 33) heart failure (HF) with multivariate analysis, also taking the etiology of cardiomyopathy into account (ischemic or non-ischemic). Statistical analysis was performed with Spearman’s correlation.

We examined the correlation between NT-proBNP, the most important prognostic marker of HF, and PACAP-38 levels. In acute HF, we did not find any significant connection between the two examined markers (*p* = 0.090, r = −0.307). On the other hand, a weak significant negative correlation was detected in the chronic HF patients (*p* = 0.049, r = −0.349). Multivariate analysis was performed also taking the etiology of the cardiomyopathy into account (ischemic or non-ischemic) showing a positive connection (*p* = 0.058, r = 0.534) between PACAP-38 and NT-proBNP in acute HF group (Figure 2A). Moreover, in chronic HF patients significantly strong negative correlation (*p* < 0.001, r = −0.746) was detected between two examined factors with multivariate analysis (Figure 2B).

### 2.3. Correlation of Plasma PACAP-38 Levels with Different Clinical and Laboratory Parameters

To examine the potential influencing factors on plasma PACAP-38 levels—comorbidities, different medical or instrumental therapeutic opportunity, echocardiographic or routine laboratory parameters—correlation and multivariate analysis tests were performed. We did not detect any significant individual or additive effect of the examined factors on the plasma PACAP-38 levels of the HF patients (Table 2).

**Figure 3:**
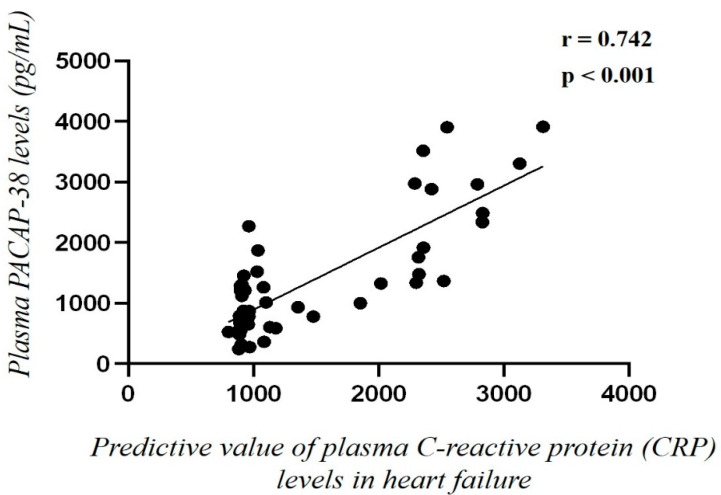
Correlation between the predictive value of C-reactive protein (CRP) (mg/mL) and plasma PACAP-38 levels (pg/mL) with multivariate analysis, also taking the type of heart failure into account (acute or chronic) (*n* = 46). Statistical analysis was performed with Spearman’s correlation.

A significant, weak positive correlation (r = 0.385, *p* = 0.001) was detected between C-reactive protein (CRP) and PACAP-38 levels in the merged (acute and chronic) HF patient group. Multivariate analysis taking into account the type of HF (acute or chronic) also showed a significant strong positive correlation (r = 0.742, *p* < 0.001) between PACAP-38 and CRP (Figure 3). The relationship between CRP and PACAP-38 revealed significant positive correlation (r = 0.615, *p* = 0.025) in acute HF patients. In the chronic group, a positive tendency (r = 0.497, *p* = 0.059) was found between these two markers.

### 2.4. Correlation of Plasma PACAP-38 Levels with Pro- and Anti-Inflammatory Cytokines

**Figure 4:**
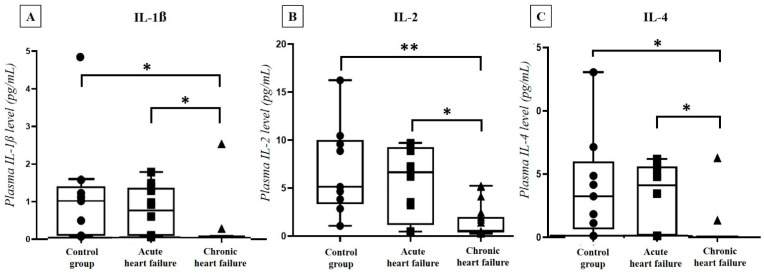
IL-1 β (**A**), IL-2 (**B**) and IL-4 (**C**) levels in acute (*n* = 12) and chronic heart failure (*n* = 19) patients and in the control group (*n* = 9). Boxes with lines and whiskers represent the interquartile range, median values and the outliers. The individual values are presented with black dots (control group), squares (acute HF) or triangles (chronic HF). Statistical analysis was performed with one-way ANOVA test with Tukey post-hoc test. * *p* < 0.05, ** *p* < 0.001 vs. chronic heart failure group.

In the HF group of 31 patients (12 acute HF, 19 chronic HF) and 9 controls we also performed a Luminex array to determine the plasma level of 7 characteristic pro- and anti-inflammatory cytokines (IL-1 β, IL-2, IL-4, IL-6, IL-10, IFN-γ, TNF-α). In the chronic HF group, significantly lower cytokine concentrations were detected compared to both the acute HF and the control groups regarding IL-1 β (acute: *p* = 0.011, control: *p* = 0.002), IL-2 (acute: *p* = 0.002, control: *p* < 0.001) and IL-4 (acute: *p* = 0.025, control: *p* = 0.004) levels (Figure 4).

**Figure 5:**
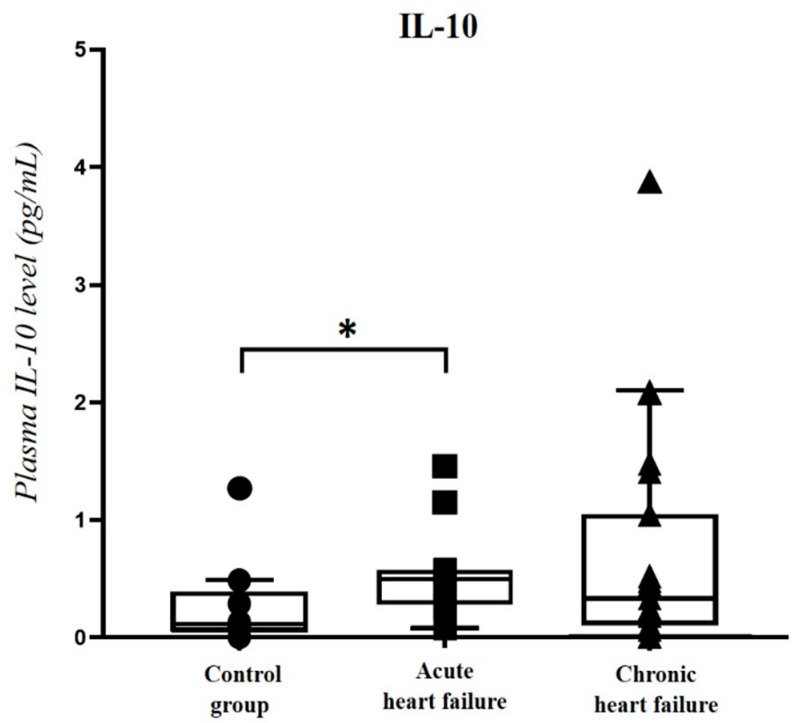
IL-10 levels in acute (*n* = 12) and chronic heart failure (*n* = 19) patients and in the control group (*n* = 9). The box plot diagram represents the interquartile range, median values and the outliers. The individual values are presented with black dots (control group), squares (acute HF) or triangles (chronic HF). Statistical analysis was performed with one-way ANOVA test with Tukey post-hoc test. * *p* < 0.05 vs. control group.

In addition, significantly (*p* = 0.038) higher IL-10 levels were detected in the acute HF group compared to the control, but not to the chronic HF group (Figure 5). In contrast, we did not detect any significant differences between the HF and control groups regarding IL-6, IFN-γ and TNF-α levels.

Furthermore, the correlation analysis showed significant positive correlation between IL-1 β, IL-2, IL-4, IL-10 cytokines and PACAP-38 levels in the HF cohort. Multivariate analysis taking the type of HF into consideration (acute/chronic) showed a more remarkable positive correlation between these cytokines and plasma PACAP-38 concentrations (Table 3).

### 2.5. Comparison of PACAP-38 Levels in Heart Tissue Lysate of Non-Ischemic Cardiomyopathy (NICM) and Ischemic Cardiomyopathy (ICM) Patients and Healthy Controls

In the second part of this study, we examined myocardial tissues of 23 advanced HF patients undergoing heart transplantation. The underlying diseases were non-ischemic cardiomyopathy (NICM) in 11 cases (47.8%) and ischemic (ICM) in 12 (52.2%) cases. The most important anthropometric and hemodynamic parameters of the patients are presented in Table 4.

**Figure 6:**
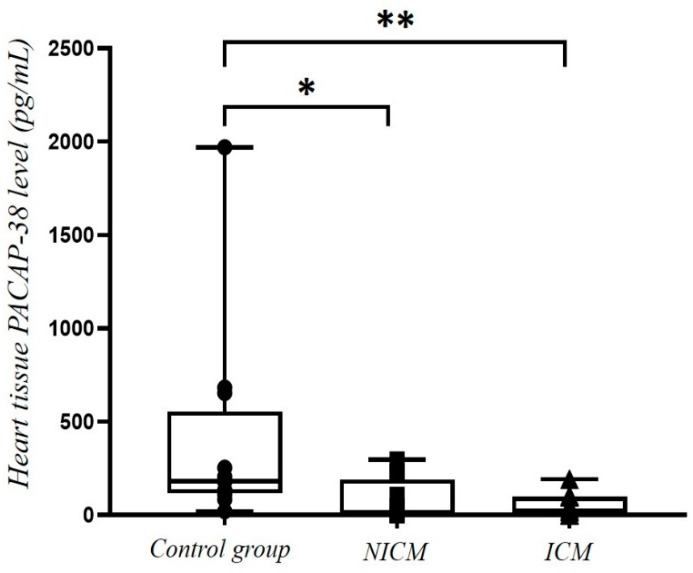
Tissue PACAP-38 levels in heart tissue samples from patients with non-ischemic cardiomyopathy (NICM, *n* = 11) or ischemic cardiomyopathy (ICM, *n* = 12) and from the healthy control group (*n* = 12). The box plot diagram represents the interquartile range, median values. The individual values are presented with black dots (control group), squares (acute HF) or triangles (chronic HF). Statistical analysis was performed with one-way ANOVA test with Tukey post-hoc test.* *p* < 0.050, ** *p* < 0.001 vs. control group.

As a control group, we also included 12 healthy organ donors (mean age: 31.11 ± 3.1 years, 50% men, 50% women, mostly dying of traffic accidents). From the homogenized myocardial tissue samples, first, we determined the tissue PACAP-38 levels, then, we compared the concentrations of the three cohorts. Significantly higher tissue PACAP-38 levels were detected in the healthy control group compared to both the NICM (*p* = 0.007) and the ICM (*p* < 0.001) group (Figure 6). There was no difference between ischemic and non-ischemic cardiomyopathy patients.

### 2.6. Correlation of Tissue PACAP-38 Levels with Different Clinical and Laboratory Parameters

Furthermore, we performed correlation analysis in the examined patient groups to reveal the potential connection between tissue PACAP-38 levels and NT-proBNP, as well as different echocardiographic and routine laboratory parameters. As indicated in Table 5, these statistical tests revealed no significant correlation between the possible influencing factors and the tissue PACAP-38 levels.

### 2.7. Comparison of PAC1 Receptor Level in the Heart Tissue Lysate of NICM and ICM Patients and Healthy Controls

**Figure 7:**
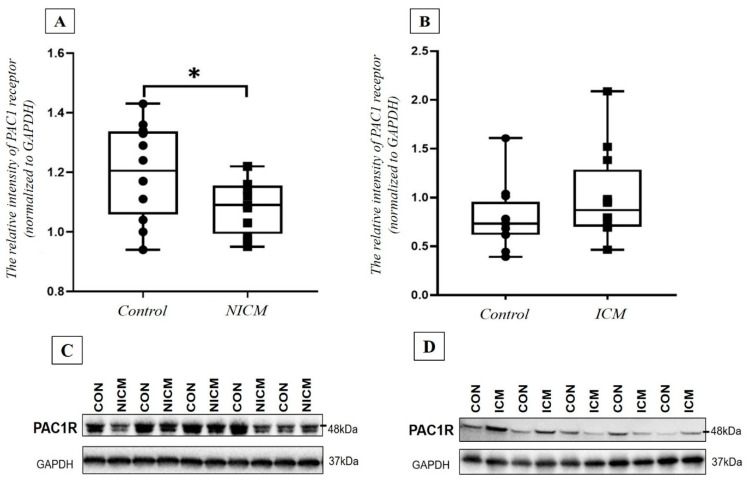
PAC1 receptor relative intensity in non-ischemic cardiomyopathy (**A**) (NICM, *n* = 11) and ischemic cardiomyopathy (**B**) (NICM, *n* = 11; ICM, *n* = 12) vs. control group (CON, *n* = 12). (**C**,**D**) pictures show the scanned Western blot representative images. PAC1 receptor values are normalized to GAPDH. The box plot diagram represents the interquartile range and median values. The individual values are presented with black dots (control group) or squares (NICM or ICM). Statistical analysis was performed with Mann–Whitney test. * *p* < 0.05 vs. control group.

Finally, the PAC1 receptor level was also examined with Western blot assay in the heart tissue samples of HF patients and control individuals. The densitometry analysis revealed significantly lower PAC1 receptor intensity in the tissue samples obtained from the NICM group compared to the healthy controls (*p* = 0.045). In contrast, there were no significant differences in the relative PAC1 receptor intensities between the ICM and the control group (*p* = 0.145) (Figure 7).

## 3. Discussion

In the current study, we provided the first evidence for significant differences between plasma PACAP-38 levels in acute and chronic HF patients and healthy controls. We also studied the potential influencing factors of plasma PACAP-38 levels. Moreover, this is the first human study examining PACAP-38 LI in myocardial tissue samples of NICM and ICM patients and also healthy controls. The significant differences between the tissue PACAP-38 levels and PAC1 receptor intensity of HF patients and controls emphasize the importance of PACAP-38 signaling in HF.

Our results showed significantly higher plasma PACAP-38 levels in acute HF patients compared to both the chronic HF and the control groups. The elevated PACAP-38 levels in acute HF can be the result of a compensating “stress response” to a suddenly worsening left ventricular ejection fraction. This reactive phenomenon seems to be a protective response, potentially decreasing acute cardiomyocyte injury. Perna and co-workers examined 115 patients with HFrEF detecting advanced cardiomyocyte injury in more than half of the patients. The extent of the cardiomyocyte injury showed strong correlation with the prognosis of HF [22]. This myocyte damage is caused by oxidative stress, apoptosis and necroptosis. PACAP effectively promotes cardiomyocytes against oxidative stress-induced apoptosis in cell culture. As a result of PACAP treatment, significantly decreased caspase-3 activity and significantly higher anti-apoptotic Bcl-2 and phospho-Bad expression were identified in cultured cardiomyocytes [9,14]. Furthermore, PACAP treatment significantly inhibited oxidative stress-induced activity of pro-apoptotic JNK and p38-MAP-kinase in endothelial cells [8]. Similar PACAP-38 release was observed during some acute severe or life-threatening human diseases, such as acute ST-segment elevation myocardial infarction [21]. Based on these findings we suppose, that PACAP-38 can be regarded as a general marker that may indicate the severity of the tissue injury, and also as a protective factor, eliciting antioxidant, antiapoptotic and anti-inflammatory effects in response to acute cellular damage [23].

In contrast, significantly lower plasma PACAP-38 levels were detected in chronic HF compared not only to the acute HF patients but also to the control group. The lower PACAP-38 levels may be involved in the diminished cardioprotective mechanisms, making the patients susceptible for further progression of HF. Mori and co-workers investigated the effects of PACAP on the progression of HF in doxorubicin-induced cardiomyopathy, observing worse prognosis of HF in PACAP-deficient heterozygous and homozygous mice compared to wild types [18]. According to their data, left ventricular dilatation was significantly higher, while ejection fraction was significantly lower in PACAP-deficent mice; moreover, the mortality rate was significantly higher, suggesting that endogenous PACAP plays an important role in cardiomyocyte protection, and indicating that a lack of PACAP indicates worse progression of HF [18]. In another in vitro model of toxic cardiomyopathy applying mitoxanrone, it was found that PACAP-38 treatment significantly decreased the damage of the left ventricular systolic function [19]. Furthermore, Otto and co-workers revealed that pulmonary hypertension and decreased right ventricular systolic function developed in PAC1 receptor-deficient mice [20], supporting this cardioprotective theory. Albeit these findings are quite convincing, further longitudinal follow-up human studies are necessary to prove that a lower plasma PACAP level is a predictive factor of worse prognosis of HF.

Regarding the connection between PACAP-38 and NT-proBNP, which is the most informative cardiac biomarker of HF, we found a remarkable significant negative correlation between their plasma levels in the chronic HF group, implying a potential role of PACAP-38 in the ethiology of cardiomyopathy. This fact confirms our assumption that low PACAP levels may be a potential biomarker of worse prognosis, such as elevated NT-proBNP levels. An increasing number of studies suggest a potential clinical use of PACAP as a diagnostic and prognostic biomarker in various pathological conditions; however, the question whether PACAP alterations are consequences or contributing factors of the disease remains open. Similar results were found in our earlier study, examining NICM and ICM patients, where we observed a significant negative correlation between plasma PACAP-38 and NT-proBNP levels in the ischemic group, but there was no significant correlation in the non-ischemic group [24]. Contrarily, we detected a positive tendency in the acute HF group between the two examined markers. NT-proBNP levels are usually elevated in acute decompensated HF due to the increased atrial wall strain caused by volume and pressure overload [25]. However, the prognostic value of NT-proBNP is weaker in the acute decompensated period before treatment compared to the compensated stable chronic HF [25].

It is a known fact that the not infection-related elevation of plasma CRP levels may mark systemic cardiac stress response and have a prognostic role in HF. The positive correlation between PACAP-38 and CRP levels are also strengthening the potential biomarker role of PACAP-38 in HF. This theory is further supported by our earlier study, where we detected sigificant positive correlation between PACAP and CRP in polytrauma patients during the acute phase [23]. However, it is important to note that circulating PACAP-38 levels alone may not be a useful biomarker for individuals suffering from HF; therefore, additional, complementary measures of other cardiac biomarkers may need to be combined with the polypeptide. In the future, it seems feasible that a combination of multiple cardiovascular biomarkers in one diagnostic panel will be used for the early diagnosis and reliable prediction of progression or therapeutic response in HF.

Examining the plasma cytokine levels in the different HF groups, we obtained diverse results. Significantly lower IL-1 β, IL-2 and IL-4 cytokine levels were detected in the chronic HF group compared to both the acute HF and the control groups. Several earlier studies proved that the baseline therapy of HF containing ACEI, β-blocker and MRA remarkably decrease the serum levels of these cytokines [5,26,27]. Moreover, some studies reported significantly decreased level of inflammatory markers in patients with CRT therapy, especially in the responder cases [27]. In this current study, the rate of the optimal medical therapy was over 80% in the chronic HF group; all of the examined patients received ACEI and β-blocker therapy and 81.8% of them were also taking MRA. Numerous previous investigations have been conducted examining the different cytokines in HF, but these result are still conflicting [28,29]. Although some have reported higher cytokine levels in HF [28,29], we assume that the lower cytokine levels can be explained by the extremely frequent application of the baseline HF therapy. Moreover, some examinations also showed that the alteration in different cytokine levels is associated with the severity of HF and the NYHA status [5,30]. They found significantly higher cytokine levels in patients with more severe HF and in NYHA stage III–IV. All our chronic HF patients had compensated cardiac status with NYHA stage I–II, which might also explain the low cytokine levels we measured in our assays.

Regarding the anti-inflammatory cytokine IL-10, we found significantly higher IL-10 levels in the acute HF group compared to the controls. Moreover, there was a significant positive correlation between IL-10 and plasma PACAP-38 levels, especially when the type of the HF (acute/chronic) was also considered. IL-10 is widely investigated in different experimental and human studies. In HF patients, significantly increased levels of plasma IL-10-secreting B cells were detected [31], suggesting that the elevated IL-10 levels may be part of the protective response in the acute decompensated HF.

Interestingly, we did not detect any significant differences in IL-6, IFN-γ and TNF-α levels between acute, chronic HF and the control groups, although several investigations discussed remarkable elevated levels of these cytokines in acute decompensated HF [27,29]. Our results fall in line with earlier controversal findings. The pathological role of IFN-γ in HF is still unclear, as the results from the limited number of clinical and animal examinations are controversial [32]. TNF-α, the most potent inflammatory cytokine, shows promising results in HF and cardiac remodeling. Several studies detected elevated circulating TNF-α levels in HFrEF correlating with worse prognosis and increased mortality. Surprisingly, clinical trials of anti-TNF-α therapy resulted in increasing all-cause mortality and HF hospitalization [33]. In addition, the above-mentioned medical treatment in HF patients may also influence the plasma level of various inflammatory cytokines, i.e., lower IL-6 concentration can be detected in HF patients after β-blocker treatment [34]. All of these conflicting results about the potential protective or harmful effects of TNF-α and IFN-γ and the unknown underlying mechanisms can explain the inconsistency between the published data and our present results.

In the second part of our study, we examined human heart tissue samples and we found conflicting results between HF groups with different etiology and control groups. Earlier, we have already shown the presence of PAC1 receptor in human cardiac tissue samples with immunohistochemistry examination [35]. Since the etiology of HF (primary or ischemic) may influence PAC1 receptor expression and the tissue levels of PACAP-38 peptide, we examined the level of these molecules in the different groups. Correlation tests showed no significant connection between the clinical or myocardial functional parameters (e.g., ejection fraction, cardiac output), the routine laboratory parameters and tissue PACAP-38 levels, indicating no influencing effect on the PACAP levels, similarly to other human studies [21].

We examined the levels of intracellular PACAP-38 in homogenates of NICM and ICM hearts and healthy myocardial tissues. Our present results revealed a significantly lower tissue PACAP level in the end-stage HF hearts compared to the healthy ones, which can be explained by our earlier data, suggesting that intracellular PACAP-38 level, or accumulation of the polypeptide, is mostly related to the living, intact cells [10,35]. In contrast with the plasma levels, we did not find any significant correlation between tissue PACAP-38 levels, plasma NT-proBNP concentrations and different echocardiographic parameters. The exact source of the tissue PACAP-38 is not known, as PACAP-38 was detected in the nerves, myocytes, extracellular matrix and also in the cytoplasm of infiltrating macrophages [36]. Based on these data, we assume that the damaged myocytes or the “exhausted” compensation mechanisms might lead to the lower tissue PACAP-38 levels that we found in end-stage HF. The latter is strengthened by literature data, showing that the natriuretic peptide levels can be extremely low in some cases of end-stage HF due to the “exhausted” neurohormonal system [37], making the correlation analyses more difficult [24].

Finally, we examined the PAC1 receptor intensity in the collected heart tissue samples. In our earlier experiment, we showed PAC1 receptor expression in the heart muscle cells; in contrast, in the endocardial connective tissue, we did not detect PAC1 receptor positivity [35]. However, in the current study, we first performed PAC1 receptor quantification using Western blot in human cardiac tissue samples, detecting significant differences between different etiological HF groups and the healthy controls. In NICM patients, significantly lower PAC1 receptor intensity was detected, while we found no significant difference in PAC1 receptor density in the ischemic group compared to the healthy controls. The possible explanation for these results is based on the different pathophysiology of the ischemic and non-ischemic cardiomyopathy. In NICM, the main underlying mechanisms are apoptosis, myocardial fibrosis and consequential cardiac remodeling [38]. We suggest that the increased cardiomyocyte apoptosis and the complex medical treatment together may lead to a decreased level of PAC1 receptors. In contrast to the above-mentioned pathomechanism, both the repeated ischemic attacks and preconditioning play an important role in ICM. The ischemic preconditioning enhances beneficial and protecting effect against ischemic injury and increases the production of various factors, such as adenosine, bradykinin or opiates [39]. In an earlier study, we detected a positive tendency between the different conditioning techniques and the plasma PACAP-38 levels [21]. However, there are no clinical data about the connection between preconditioning and PAC1 receptor level. Based on experimental and our human study results, we hypothesized that the relatively higher PAC1 receptor intensity is caused by the ischemic preconditioning in the ICM group. The presence of PAC1 receptors in myocardium raises the possibility of therapeutic use of endogenous or exogenous PACAP taking advantage of the anti-apoptotic, anti-inflammatory and anti-oxidant properties.

## 4. Materials and Methods

### 4.1. Plasma and Serum Samples of HF Patients

In the first part of our study, 13 patients with acute decompensated HF (mean age: 66.5 ± 3.7 years, 33% women, 77% men), 33 patients with chronic, compensated HF (mean age: 65.9 ± 3.8 years, 34.3% women, 75.7% men) and 13 age- and gender-matched controls without HF (mean age: 65.8 ± 4.0 years, 31% women, 69% men) were examined. Patients with ICM and NICM were included in the acute HF group, admitted to intensive care unit due to reduced ejection fraction (EF < 40%) and symptoms of acute cardiac decompensation (shortness of breath, limitation of physical activity, NYHA III–IV stage). In contrast, patients with compensated chronic HF due to dilated cardiomyopathy were enrolled in the chronic HF group. Their cardiovascular status was compensated (NYHA II stage) and patients were on stable pharmacological treatment at least 3 months prior the enrollment. We also involved an age- and gender-matched control group in our study, including patients examined in our hospital for hypertension or atypical chest pain without symptoms or evidence of HF. Structural heart disease, impaired left ventricular systolic function (EF > 55%) and ischemic heart diseases were excluded by coronarography or coronary CT angiography. All patients possessing any inflammatory disease were excluded from the study.

Several routine laboratory tests were performed in all participants: three tubes (native, EDTA (ethylenediaminetetraacetic acid) and citrate tubes) were taken for general laboratory testing. Inflammatory parameters (serum CRP level), renal function (serum creatinine and urea levels), complete blood count and lipid parameters (serum total cholesterol, LDL cholesterol, HDL cholesterol, and levels of triglycerides) were determined. NT-proBNP measurements were also performed in cases of acute decompensated HF and chronic HF. All laboratory tests were performed in the Department of Laboratory Medicine, Clinical Center, University of Pecs. For detection of PACAP-38 LI, another 10 mL tube of peripheral venous blood including EDTA was also taken. Due to the polypeptide nature of PACAP-38, a protease inhibitor (200 µL aprotinin (stock 1.4 mg/mL) into 10 mL blood) was added to the blood samples and an ice water bath was used for storing the tubes to avoid peptide degradation. The EDTA-tubes were centrifuged immediately after the collection (4000 rpm, 4 °C, 15 min), after which the supernatant was collected and stored at −80 °C in polypropylene tubes (Sarstedt, Budapest, Hungary), then PACAP-38 sandwich-type enzyme-linked immunosorbent assay (ELISA) and cytokine analysis with Luminex array were performed using these samples.

All human sample collections were carried out according to a protocol approved by the Institutional Ethics Committee (PTE KK 6383). In all cases, we obtained informed consent of the volunteers.

### 4.2. Cardiac Tissue Samples of HF Patients

Human heart samples were collected in the Department of Heart Failure and Transplantology, Cardinal Stefan Wyszyński National Institute of Cardiology, Warszawa, Poland, as previously described [40]. Healthy human hearts were obtained from organ donor patients (control, *n* = 12). The donors did not have any relevant previous cardiological history or any abnormalities in ECG and echocardiography (LV dimensions/contractility within normal ranges), and the control hearts were not used for transplantation due to technical reasons (e.g., due to donor/recipient incompatibility). Explanted failing hearts were obtained from patients suffering from advanced HF of non-ischemic (NICM, *n* = 11) or ischemic (ICM, *n* = 12) etiology. Human left ventricular tissue samples were taken from free wall, at the time of heart explantation (avoiding scarred, fibrotic or adipose tissue, endocardium, epicardium or coronary vessels). The samples were rinsed immediately in physiological saline, blotted dry, frozen in liquid nitrogen and kept at −80 °C until further processing.

For tissue disruption, a total of 30 mg frozen cardiac tissue samples were sonicated with a Hielscher UP 200 H/S homogenizer (Hielscher Ultrasonics GmbH, Teltow, Germany) in 500 μL of ice-cold phosphate buffered saline (PBS) containing 14 μg aprotinin as protease inhibitor. Sonication was performed on ice with 3 × 30 s bursts and an amplitude of 30%. Then, the homogenates were centrifuged at 10,000 rpm, for 15 min at 4 °C. The obtained supernatants were collected and tested for PACAP-38 LI with a PACAP-38-specific ELISA.

All experimental procedures were done in accordance with the ethical standards of the responsible institutional and national committee on human experimentation, adhering to the Helsinki Declaration (1975). Written informed consent was obtained from all patients involved in the study according to the protocol approved by the Local Ethics Committees of the Institute of Cardiology, Warszawa, Poland (IK-NP-0021-24/1426/18).

### 4.3. Measurement of PACAP-38 Like Immunoreactivity (LI) by ELISA

For the determination of PACAP-38 LI in human cardiac tissue homogenates and plasma samples, sandwich-type enzyme-linked immunosorbent assay (human PACAP-38 ELISA kit, MyBiosource, San Diego, CA, USA, cat.No: MBS109020) was used according to the protocol provided by the manufacturer. PACAP-38 LI is referred to as PACAP-38 level in the manuscript. Briefly, 50 μL of PACAP-38 standards, tissue homogenates, and plasma of myocardial infarction patients and healthy controls were pipetted to the appropriate wells of the anti-PACAP-38 antibody-precoated microwells in duplicate. Then, 100 μL of horseradish peroxidase (HRP)-conjugated reagent was added to each well, covered with closure plate and incubated for 60 min at 37 °C. The plate was washed four times with 200 μL/well of 1× Wash buffer. Next, 50 μL of Chromogen Solution A and 50 μL of Chromogen Solution B was added to each well and incubated for 15 min at 37 °C in dark. The developing color reaction was stopped by adding 50 μL of Stop solution to every well. The SPECTROStar Nano spectrophotometer (BMG Labtech, Ortenberg, Germany) was used to measure the optical density (OD) of the test-wells at a wavelength of 450 nm. Since the obtained OD values were proportional to the level of PACAP-38 in the test samples, their concentrations were calculated by comparing the OD values of the sample wells to the ODs of the standard curve. All measured plasma PACAP-38 levels are shown in pg/mL.

### 4.4. Measurement of Pro- and Anti-Inflammatory Cytokine Levels with LUMINEX Array

Plasma samples from 9 healthy control and 31 HF patients (*n* = 12 acute HF, *n* = 19 chronic HF) were examined for the concentrations of 7 characteristic pro-, and anti-inflammatory cytokines (IL-1 β, IL-2, IL-4, IL-6, IL-10, IFN-g, TNF-a) with the high sensitivity Invitrogen™ Human Cytokine 7-Plex ProcartaPlex™ Panel (Thermo Fisher Scientific, Vienna, Austria) according to the manufacturer’s instructions. All tests were run in duplicate. Briefly, first, the kit components were allowed to warm up to room temperature. Then, plasma samples were thawed and 25 µL volume/well plasma and diluted cytokine standards were loaded onto a 96-well plate containing 25 µL of capture antibody-coated fluorescent-coded beads. The plate was incubated for 30 min. After washing, 25 µL biotinylated detection antibodies and 50 µL streptavidin-PE were added to the plate with alternate incubation and washing steps. After final washing 120 µL Reading buffer was added to the wells and the plate was read on the Luminex MagPix array reader. Five-parameter logistic (PL) regression curve was used to plot the 7-pt standard curves for all analytes. Data were analyzed using the Belysa 1.1.0 (Merck KGaA; Darmstadt, Germany) software. The calculated cytokine concentrations were given in pg/mL.

### 4.5. Measurement of PAC1 Receptor Level by Western Blot Analysis

In order to investigate whether PAC1 receptor level is altered at the protein level in the homogenates of heart samples, Western blot was performed as previously described in our laboratory with modifications [41]. Frozen tissue samples were homogenized in 1× radio immunoprecipitation assay buffer (RIPA; Cell Signaling Technology, Danvers, MA, USA), supplemented with 1× HALT Protease and Phosphatase Inhibitor cocktail (Thermo Scientific, Waltham, MA, USA). Protein concentration of the samples was determined by bicinchoninic acid assay kit (Thermo Scientific, Waltham, MA, USA). Equal amounts of protein from each sample were mixed with 1/4th total volume of Laemmli buffer containing β-mercaptoethanol (Thermo Scientific, Waltham, MA, USA) and were loaded on 4–20% Tris-glycine sodium dodecyl sulfate-polyacrylamide gradient gels (Bio-Rad, Hercules, CA, USA), and electrophoresed at constant voltage (on 90 V for 15 min and on 110 V for 1.5 h, room temperature). The separated proteins were transferred onto polyvinylidene difluoride membrane (Bio-Rad, Hercules, CA, USA) with Trans-Blot^®^ Turbo™ Transfer System (2.5 A, 7 min, room temperature, Bio-Rad, Hercules, CA, USA). Next, membranes were blocked with 5% bovine serum albumin (Bio-Rad, Hercules, CA, USA) in Tris-buffered saline containing 0.05% Tween-20 (0.05% TBS-T; Sigma, St. Louis, MO, USA) for 2 h at room temperature, and then were probed with primary antibodies overnight at 4 °C (anti-ADCYAP1R1/PAC1: 1:2500, cat. No: SAB2900693, Sigma, St. Louis, MO, US; GAPDH: 1:5000, cat. No: 2118, Cell Signaling, Danvers, MA, USA). After a 3 × 10-min wash in 0.05% TBS- T, membranes were incubated with corresponding HRP-conjugated secondary antibodies (anti-rabbit: 1:5000, cat. No: 7074; Cell Signaling, Danvers, MA, USA) for 2 h at room temperature and washed in 0.05% TBS-T again for 3 × 10 min. Signals were visualized after incubation with Clarity^TM^ Western ECL Substrate chemiluminescence kit (#170506S; Bio-Rad, Hercules, CA, USA) by Chemidoc XRS+ Gen Imagine System (Bio-Rad, Hercules, CA, USA). Image analysis was performed using Image Lab™ 6.0 software (Bio-Rad, Hercules, CA, USA). The measured density of PAC1 receptor was normalized to the intensity of GAPDH specific lane and presented as relative PAC1 receptor intensity.

### 4.6. Statistical Analysis

For statistical analysis, SPSS 22 (Statistical Package for the Social Sciences, Chicago, IL, USA) Program was used. Kolmogorov–Smirnov and Shapiro–Wilk normality tests were performed, showing normally distributed data. To detect the potential differences between the examined groups (acute or chronic HF vs. control; ICM or NICM vs. control), one-way ANOVA with Tukey post hoc tests were used. Mann–Whitney test was performed to examine the differences in PAC1 receptor intensity between the different cardiomyopathy groups (ICM or NICM) and healthy controls. The interaction between PACAP-38 and NT-proBNP, CRP, different cytokine levels and other potential impacting factors (comorbidities, echocardiographic parameters, therapy and routine laboratory parameters) were tested with Spearman’s correlation. Based on the correlation coefficient (the *r* value), we could define positive (r = 0–1) and negative (r = −1–0) correlation, including subgroups with different strength. Multivariate regression analysis was performed to examine the additive effects of the main influencing factors. In all cases, *p* < 0.05 was considered statistically significant.

## 5. Conclusions

In this study, we detected significantly higher plasma PACAP-38 levels in acute, decompensated HF and significantly lower PACAP-38 in chronic, compensated HF compared to the healthy control group. PACAP-38 also showed strong correlations with important HF biomarkers, such as NT-proBNP and CRP. Moreover, we revealed significant correlation between PACAP-38 and different pro- and anti-inflammatory cytokines. Furthermore, significantly higher tissue PACAP-38 was detected in the healthy controls compared to both ischemic and non-ischemic cardiomyopathies. Additionally, we found significantly lower PAC1 receptor intensity in the NICM group compared to the controls. All of these results highlight the importance and necessity to investigate the predictive biomarker role of PACAP-38 in human follow-up studies of HF patients.

## Figures and Tables

**Table 1:** The most important demographic and clinical parameters of the examined patients with acute, chronic heart failure (HF) and the control group. NYHA: New York Heart Association Classification, ACEI: angiotensin-converting enzyme inhibitor, ARB: angiotensin-II receptor blocker, MRA: mineralocorticoid receptor antagonist.

	Acute HF (*n* = 13)	Chronic HF (*n* = 33)	Control Group (*n* = 13)
Mean age (year)	66.5 ± 3.7	65.9 ± 3.8	65.8 ± 4.0
Gender	33% women 67% men	34.3% women 65.7% men	31% women 69% men
Mean ejection fraction (%)	33.1%	30.3%	38.5%
NYHA stage	III–IV. st.	II. st.	I. st.
Cardiovascular status	decompensated	compensated	no heart failure
Comorbidities			
Hypertension	69.2%	87.9%	46.1%
Diabetes mellitus	46.2%	42.4%	15.4%
Atrial fibrillation	53.8%	36.4%	7.7%
Medical therapy			
ACEI/ARB	100%	100%	38.5%
β-blocker	100%	100%	38.5%
MRA	76.9%	81.8%	0%
Diuretics	92.3%	84.8%	15.4%
Ivabradine	23.1%	9.0%	0%
Digoxin	15.4%	15.2%	0%

**Table 2:** Potential influencing factors on plasma PACAP-38 levels. ACEI: angiotensin-converting enzyme inhibitor, ARB: angiotensin-II receptor blocker, MRA: mineralocorticoid receptor antagonist, CRT: cardiac resynchronization therapy, ICD: implantable cardioverter-defibrillator, EF: ejection fraction, LV-EDD: left ventricular end-diastolic diameter, RV-EDD: right ventricular end-diastolic diameter, IVC: inferior vena cava size, LDL: low-density lipoprotein, HDL: high-density lipoprotein. Spearman’s correlation test was performed.

	Correlation Coefficient (r)	Significance (*p*)
Comorbidities		
Hypertension	r = −0.095	*p* = 0.532
Diabetes mellitus	r = 0.003	*p* = 0.983
Atrial fibrillation	r = 0.064	*p* = 0.671
Therapy		
ACEI/ARB	-	-
β-blocker	-	-
MRA	r = 0.031	*p* = 0.178
Diuretics	r = 0.081	*p* = 0.708
Ivabradin	r = 0.206	*p* = 0.326
Digoxin	r = 0.048	*p* = 0.822
CRT	r = 0.005	*p* = 0.973
ICD	r = 0.067	*p* = 0.659
Echocardiographic parameters		
EF (%)	r = 0.113	*p* = 0.456
LV-EDD (mm)	r = 0.063	*p* = 0.689
RV-EDD (mm)	r = −0.012	*p* = 0.938
Mitral regurgitation	r = 0.045	*p* = 0.776
Tricuspid regurgitation	r = 0.034	*p* = 0.827
IVC (mm)	r = 0.067	*p* = 0.671
Laboratory parameters		
Cholesterol	r = 0.043	*p* = 0.736
LDL cholesterol	r = 0.183	*p* = 0.474
HDL cholesterol	r = 0.041	*p* = 0.826
Triglycerides	r = 0.033	*p* = 0.354
Blood urea nitrogen	r = 0.010	*p* = 0.946
Serum creatinine	r = 0.100	*p* = 0.514

**Table 3:** Correlation between the plasma level of different cytokines (IL-1 β, IL-2, IL-4 and IL-10) and PACAP-38 with Spearman’s correlation test or with multivariate analysis (taking also into account the type of heart failure: acute or chronic). Statistically significant differences with *p*-values of ** *p* < 0.001 and * *p* < 0.05 are indicated.

Cytokines	Correlation Test		Multivariate Analysis	
	Correlation Coefficient (r)	Significance (*p*)	Correlation Coefficient (r)	Significance (*p*)
IL-1 β	r = 0.539 *	*p* = 0.002	r = 0.780 **	*p* < 0.001
IL-2	r = 0.494 *	*p* = 0.005	r = 0.812 **	*p* < 0.001
IL-4	r = 0.481 *	*p* = 0.006	r = 0.800 **	*p* < 0.001
IL-10	r = 0.367 *	*p* = 0.042	r = 0.799 **	*p* < 0.001

**Table 4:** The most important anthropometric data, echocardiographic and hemodynamic parameters of the examined patients with non-ischemic cardiomyopathy (NICM) and ischemic cardiomyopathy (ICM) before heart transplantation.

	NICM (*n* = 11)	ICM (*n* = 12)
Mean age (years)	39.18 ± 3.4	59.42 ± 3.8
Gender	90.9% men 9.1% women	91.7% men 8.3% women
Echocardiographic parameters		
End-diastolic diameter (mm)	75.45 ± 3.1	72.92 ± 1.9
End-systolic diameter (mm)	67.34 ± 2.9	65.75 ± 1.8
Ejection fraction (%)	17.09 ± 1.4	21.92 ± 2.3
Hemodynamic parameters		
Cardiac output (L/min)	4.03 ± 0.22	4.28 ± 0.56
Mean heart rate (bpm)	104.5 ± 3.1	74.33 ± 4.9
Mean systolic blood pressure (mmHg)	99.56 ± 3.4	107.17 ± 4.9
Mean diastolic blood pressure (mmHg)	62.89 ± 4.1	56.58 ± 4.6

**Table 5:** Examination of the correlation between the potential influencing factors and tissue PACAP-38 levels. NICM: non-ischemic cardiomyopathy, ICM: ischemic cardiomyopathy, EF: ejection fraction, LV-EDD: left ventricular end-diastolic diameter, RV-EDD: right ventricular end-diastolic diameter, LDL: low-density lipoprotein, HDL: high-density lipoprotein. Spearman’s correlation test was used to examine the correlation between NT-proBNP levels, different echocardiographic and laboratory parameters and heart tissue PACAP-38 levels. Moreover, multivariate analysis was performed, also considering the etiology of cardiomyopathy (non-ischemic or ischemic).

	Correlation Coefficient (r)	Significance (*p*)
NT-proBNP (pg/mL)		
All patients	r = −0.167	*p* = 0.435
NICM	r = −0.041	*p* = 0.899
ICM	r = −0.254	*p* = 0.425
with multivariate analysis	r = −0.187	*p* = 0.688
Echocardiographic parameters		
EF (%)	r = 0.146	*p* = 0.494
LV-EDD (mm)	r = 0.167	*p* = 0.369
RV-EDD (mm)	r = −0.177	*p* = 0.407
Posterior wall thickness (mm)	r = 0.240	*p* = 0.451
septal wall thickness (mm)	r = 0.197	*p* = 0.540
Routine laboratory tests		
Cholesterol	r = 0.068	*p* = 0.751
LDL cholesterol	r = 0.089	*p* = 0.693
HDL cholesterol	r = 0.057	*p* = 0.766
Triglycerides	r = 0.129	*p* = 0.567
Blood urea nitrogen	r = −0.031	*p* = 0.887
Creatinine	r = −0.122	*p* = 0.578
Sodium	r = −0.280	*p* = 0.196
Potassium	r = −0.307	*p* = 0.154

## Data Availability

Not applicable.

## References

[1] Huang Y.T., Tseng Y.T., Chu T.W., Chen J., Lai M.Y., Tang W.R., Shiao C.C. (2016). *N*-terminal pro b-type natriuretic peptide (NT-pro-BNP)-based score can predict in-hospital mortality in patients with heart failure. Sci. Rep..

[2] Taylor K.S., Verbakel J.Y., Feakins B.G., Price C.P., Perera R., Bankhead C., Pluddemann A. (2018). Diagnostic accuracy of point-of-care natriuretic peptide testing for chronic heart failure in ambulatory care: Systematic review and meta-analysis. BMJ.

[3] Pan W., Yang D., Yu P., Yu H. (2020). Comparison of predictive value of NT-proBNP, sST2 and MMPs in heart failure patients with different ejection fractions. BMC Cardiovasc. Disord..

[4] Hedayat M., Mahmoudi M.J., Rose N.R., Rezaei N. (2010). Proinflammatory cytokines in heart failure: Double-edged swords. Heart Fail. Rev..

[5] Zhang Y., Bauersachs J., Langer H.F. (2017). Immune mechanisms in heart failure. Eur. J. Heart Fail..

[6] Vaudry D., Falluel-Morel A., Bourgault S., Basille M., Burel D., Wurtz O., Fournier A., Chow B.K., Hashimoto H., Galas L. (2009). Pituitary adenylate cyclase-activating polypeptide and its receptors: 20 years after the discovery. Pharmacol. Rev..

[7] Bian N., Du G., Ip M.F., Ding J., Chang Q., Li Z. (2017). Pituitary adenylate cyclase-activating polypeptide attenuates tumor necrosis factor-α-induced apoptosis in endothelial colony-forming cells. Biomed. Rep..

[8] Racz B., Gasz B., Borsiczky B., Gallyas F., Tamas A., Jozsa R., Lubics A., Kiss P., Roth E., Ferencz A. (2007). Protective effects of pituitary adenylate cyclase activating polypeptide in endothelial cells against oxidative stress-induced apoptosis. Gen. Comp. Endocrinol..

[9] Racz B., Gasz B., Gallyas F., Kiss P., Tamas A., Szanto Z., Lubics A., Lengvari I., Toth G., Hegyi O. (2008). PKA-Bad-14-3-3 and Akt-Bad-14-3-3 signaling pathways are involved in the protective effects of PACAP against ischemia/reperfusion-induced cardiomyocyte apoptosis. Regul. Pept..

[10] Reglodi D., Tamas A. (2016). Pituitary Adenylate Cyclase Activating Polypeptide–PACAP.

[11] Roth E., Weber G., Kiss P., Horvath G., Toth G., Gasz B., Ferencz A., Gallyas F., Reglodi D., Racz B. (2009). Effects of PACAP and preconditioning against ischemia/reperfusion-induced cardiomyocyte apoptosis in vitro. Ann. N. Y. Acad. Sci..

[12] Seaborn T., Masmoudi-Kouli O., Fournier A., Vaudry H., Vaudry D. (2011). Protective effects of pituitary adenylate cyclase-activating polypeptide (PACAP) against apoptosis. Curr. Pharm. Des..

[13] Sano H., Miyata A., Horio T., Nishikimi T., Matsuo H., Kangawa K. (2002). The effect of pituitary adenylate cyclase activating polypeptide on cultured rat cardiocytes as a cardioprotective factor. Regul. Pept..

[14] Gasz B., Racz B., Roth E., Borsiczky B., Tamas A., Boronkai A., Gallyas F., Toth G., Reglodi D. (2006). PACAP inhibits oxidative stress-induced activation of MAP kinase-dependent apoptotic pathway in cultured cardiomyocytes. Ann. N. Y. Acad. Sci..

[15] McDonagh T.A., Metra M., Adamo M., Gardner R.S., Baumbach A., Bohm M., Burri H., Butler J., Čelutkienė J., Chioncel O. (2021). 2021 ESC Guidelines for the diagnosis and treatment of acute and chronic heart failure. Eur. Heart J..

[16] Szobi A., Gonçalvesova E., Varga Z.V., Leszek P., Kusmierczyk M., Hulman M., Kyselovič J., Ferdinandy P., Adameova A. (2017). Analysis of necroptotic proteins in failing human hearts. J. Transl. Med..

[17] Songbo M., Lang H., Xinyong C., Bin X., Ping Z., Liang S. (2019). Oxidative stress injury in doxorubicin-induced cardiotoxicity. Toxicol. Lett..

[18] Mori H., Nakamachi T., Ohtaki H., Yofu S., Sato A., Endo K., Iso Y., Suzuki H., Takeyama Y., Shintani N. (2010). Cardioprotective effect of endogenous pituitary adenylate cyclase activating polypeptide on doxorubicin-induced cardiomyopathy in mice. Circ. J..

[19] Subramaniam V., Chuang G., Xia H., Burn B., Bradley J., Maderdrut J.L., Coy D.H., Varner K.J. (2017). Pituitary adenylate cyclase-activating polypeptide (PACAP) protects against mitoxantrone-induced cardiac injury in mice. Peptides.

[20] Otto C., Hein L., Brede M., Jahns R., Engelhardt S., Grone H.J., Schutz G. (2004). Pulmonary hypertension and right heart failure in pituitary adenylate cyclase-activating polypeptide type I receptor-deficient mice. Circulation.

[21] Szabo D., Sarszegi Z., Polgar B., Saghy E., Nemeth A., Reglodi D., Makkos A., Gorbe A., Helyes Z., Ferdinandy P. (2021). PACAP-38 in acute ST-segment elevation myocardial infarction in humans and pigs: A translational study. Int. J. Mol. Sci..

[22] Perna E.R., Macin S.M., Canella J.P., Augier N., Stival J.L., Cialzeta J.R., Pitzus A.E., Garcia E.H., Obregon R., Brizuela M. (2004). Ongoing myocardial injury in stable severe heart failure: Value of cardiac troponin T monitoring for high-risk patient identification. Circulation.

[23] Tamas A., Toth D., Pham D., Loibl C., Rendeki S., Csontos C., Rozanovic M., Bogar L., Polgar B., Nemeth J. (2021). Changes of pituitary adenylate cyclase activating polypeptide (PACAP-38) level in polytrauma patients in the early post-traumatic period. Peptides.

[24] Sarszegi Z., Szabo D., Gaszner B., Konyi A., Reglodi D., Nemeth J., Lelesz B., Polgar B., Jungling A., Tamas A. (2019). Examination of pituitary adenylate cyclase-activating polypeptide (PACAP-38) as a potential biomarker in heart failure patients. J. Mol. Neurosci..

[25] Mueller C., McDonald K., de Boer R.A., Maisel A., Cleland J., Kozhuharov N., Coats A., Metra M., Mebazaa A., Ruschitzka F. (2019). Heart Failure Association of the European Society of Cardiology practical guidance on the use of natriuretic peptide concentrations. Eur. J. Heart Fail..

[26] Gage J.R., Fonarow G., Hamilton M., Widawski M., Martínez-Maza O., Vredevoe D.L. (2004). Beta blocker and angiotensin-converting enzyme inhibitor therapy is associated with decreased Th1/Th2 cytokine ratios and inflammatory cytokine production in patients with chronic heart failure. Neuroimmunomodulation.

[27] Lappegård K.T., Bjørnstad H., Mollnes T.E., Hovland A. (2015). Effect of cardiac resynchronization therapy on inflammation in congestive heart failure: A review. Scand. J. Immunol..

[28] Bartekova M., Radosinska J., Jelemensky M., Dhalla N.S. (2018). Role of cytokines and inflammation in heart function during health and disease. Heart Fail. Rev..

[29] Hanna A., Frangogiannis N.G. (2020). Inflammatory cytokines and chemokines as therapeutic targets in heart failure. Cardiovasc. Drugs Ther..

[30] Ji Y., Ge J., Li X. (2018). Association of IL-1β polymorphisms and plasma levels with chronic heart failure: A case-control study in Chinese patients. Eur. J. Infl..

[31] Guo Y., Cen Z., Wei B., Wu W., Zhou Q. (2015). Increased circulating interleukin 10-secreting B cells in patients with dilated cardiomyopathy. Int. J. Clin. Exp. Pathol..

[32] Levick S.P., Goldspink P.H. (2014). Could interferon-gamma be a therapeutic target for treating heart failure?. Heart Fail. Rev..

[33] Chung E.S., Packer M., Lo K.H., Fasanmade A.A., Willerson J.T. (2003). Anti-TNF therapy against congestive heart failure investigators. Randomized, double-blind, placebo-controlled, pilot trial of infliximab, a chimeric monoclonal antibody to tumor necrosis factor-alpha, in patients with moderate-to-severe heart failure: Results of the anti-TNF Therapy Against Congestive Heart Failure (ATTACH) trial. Circulation.

[34] Matsumura T., Tsushima K., Ohtaki E., Misu K., Tohbaru T., Asano R., Nagayama M., Kitahara K., Umemura J., Sumiyoshi T. (2002). Effects of carvedilol on plasma levels of interleukin-6 and tumor necrosis factor-alpha in nine patients with dilated cardiomyopathy. J. Cardiol..

[35] Szanto Z., Sarszegi Z., Reglodi D., Nemeth J., Szabadfi K., Kiss P., Varga A., Banki E., Csanaky K., Gaszner B. (2012). PACAP-38 immunoreactivity in human malignant tumor samples and cardiac diseases. J. Mol. Neurosci..

[36] Alston E.N., Parrish D.C., Hasan W., Tharp K., Pahlmeyer L., Habecker B.A. (2011). Cardiac ischemia-reperfusion regulates sympathetic neuropeptide expression through gp130-dependent and independent mechanisms. Neuropeptides.

[37] Miller W.L., Hartman A.K., Burritt M.F., Burnett J.C., Jaffe A.S. (2004). Mortality in end stage heart failure is associated with paradoxically low NT-pro BNP and BNP levels: “natriuretic peptide exhaustion”?. J. Card. Fail..

[38] Dadson K., Hauck L., Billia F. (2017). Molecular mechanisms in cardiomyopathy. Clin. Sci..

[39] Tomai F., Crea F., Chiariello L., Gioffrè P.A. (1999). Ischemic preconditioning in humans: Models, mediators, and clinical relevance. Circulation.

[40] Varga Z.V., Pipicz M., Baan J.A., Baranyai T., Koncsos G., Leszek P., Kuśmierczyk M., Sánchez-Cabo F., García-Pavía P., Brenner G.J. (2017). Alternative splicing of NOX4 in the failing human heart. Front. Physiol..

[41] Baranyai T., Herczeg K., Onodi Z., Voszka I., Modos K., Marton N., Nagy G., Mager I., Wood M.J., El Andaloussi S. (2015). Isolation of exosomes from blood plasma: Qualitative and quantitative comparison of ultracentrifugation and size exclusion chromatography methods. PLoS ONE.

